# Standardized mounting method of (zebrafish) embryos using a 3D-printed stamp for high-content, semi-automated confocal imaging

**DOI:** 10.1186/s12896-019-0558-y

**Published:** 2019-10-22

**Authors:** David Simon Kleinhans, Virginie Lecaudey

**Affiliations:** Department of Developmental Biology of Vertebrates, Institute for Cell biology and Neuroscience, Goethe University, Max-von-Laue-Str. 13, 60438 Frankfurt am Main, Germany

**Keywords:** Zebrafish, Reproducibility, Standardization, Automation, Quantitative imaging, High content imaging, 3D printed stamp, Multidimensional imaging

## Abstract

**Background:**

Developmental biology relies to a large extent on the observation and comparison of phenotypic traits through time using high resolution microscopes. In this context, transparent model organisms such as the zebrafish *Danio rerio* in which developing tissues and organs can be easily observed and imaged using fluorescent proteins have become very popular. One limiting factor however is the acquisition of a sufficient amount of data, in standardized and reproducible conditions, to allow robust quantitative analysis. One way to improve this is by developing mounting methods to increase the number of embryos that can be imaged simultaneously in near-to-identical orientation.

**Results:**

Here we present an improved mounting method allowing semi-automated and high-content imaging of zebrafish embryos. It is based on a 3D-printed stamp which is used to create a 2D coordinate system of multiple μ-wells in an agarose cast. Each μ-well models a negative of the average zebrafish embryo morphology between 22 and 96 h-post-fertilization. Due to this standardized and reproducible arrangement, it is possible to define a custom well plate in the respective imaging software that allows for a semi-automated imaging process. Furthermore, the improvement in Z-orientation significantly reduces post-processing and improves comparability of volumetric data while reducing light exposure and thus photo-bleaching and photo-toxicity, and improving signal-to-noise ratio (SNR).

**Conclusions:**

We present here a new method that allows to standardize and improve mounting and imaging of embryos. The 3D-printed stamp creates a 2D coordinate system of μ-wells in an agarose cast thus standardizing specimen mounting and allowing high-content imaging of up to 44 live or mounted zebrafish embryos simultaneously in a semi-automated, well-plate like manner on inverted confocal microscopes. In summary, image data quality and acquisition efficiency (amount of data per time) are significantly improved. The latter might also be crucial when using the services of a microscopy facility.

## Background

Understanding how an organism develops from a unique, fertilized egg is largely based on observations. This includes observations at all scales to detect changes at the level of the whole organism, organs, tissue, cells and molecules. Recording data of biological processes in the mm to nm scale requires specialized and appropriate instruments that can magnify such small structures – the microscope. Therefore, since the emergence of microscopes, the fields of developmental biology and bioimaging have been developing in a coordinated manner [1–3]. Recent technological advances in microscopy, e.g. confocal spinning-disc and light-sheet microscopy, now allow for data acquisition with high spatiotemporal resolution, reduced phototoxicity and photobleaching [4].

Developmental biology evolved from a discipline largely based on qualitative methods. Due to always better microscopes, image analysis methods and computer technology, it is in a process of digital and quantitative transformation [5–8]. Given the noisy and variable character of biological systems and possibly small effect size, it is important to record a sufficient number of samples to obtain a quantitative and representative view of a biological process. Furthermore, to process biological samples of whole organisms in a high-content manner, it is important to have a standardized way of sample mounting, data acquisition, data processing and analysis. Therefore, high-resolution imaging of multiple samples in standardized conditions is of key importance.

As high-resolution imaging and quantitative biology became more and more important for developmental biologists, the zebrafish *Danio rerio* was established as an ideal model organism for vertebrate development [9–13]. Beside its genetic tractability and robustness, two of its main advantages in respect to modern light microscopy and bio-image data analysis are (i) the transparency of the embryo making it ideal for live imaging and (ii) the high number of off-spring enabling high-content screening.

To get the most of these two advantages, it is however necessary to mount, image and analyse as many embryos as possible simultaneously and in conditions that are as similar as possible. To date, this is still largely limited by the classical way most scientists mount embryos for imaging. The factors limiting this standardization are summarized in Table 1 (left column).

**Table 1:** Limitations of traditional zebrafish mounting techniques

Standard method	Limitations	Solutions	Improvement
Mounting in 1% low-melting point agarose (LMPA)	Polymerization speed limits the number of embryos that can be mounted in parallel Embryo growth is restricted by high-percentage LMPA	Use lower concentration of LMPA Use heating device to keep the LMPA liquid longer	• Mounting time extended •Growth and change in shape of the embryo allowed • Sample size increased • Embryo retrieval facilitated • Possibility to grow embryos to adulthood afterwards
No pre-defined positions	Positioning and alignment in XY are neither standardized nor reproducible	Mount embryos at pre-defined, identically oriented, equidistant positions (Figs. 1c, and 2d, f)	• Relative positions of embryos identical in all experiments • Easier setup of multi-dimensional imaging experiments • Easier navigation between different XY locations • Possibility for semi-automated imaging • Identification of individual embryo facilitated for downstream experiments (genotyping)
No μ-wells that model the average embryo shape at a defined developmental stage	Time-consuming orientation of the embryos Body axes not aligned to optical sectioning in Z due to the huge yolk sac (Fig. 1a)	Use μ-wells that model the average embryo shape (Fig. 1a’)	• Orientation of individual embryos during mounting in LMPA much faster • No need to re-orient the embryos in XY plane post-imaging • Aligned morphological shapes in Z projections • Reduced stack and file size •Reduced photo-bleaching and -toxicity • Reduced post-processing • Increased scanning speed • Improved signal-to-noise ratio

**Fig. 1:**
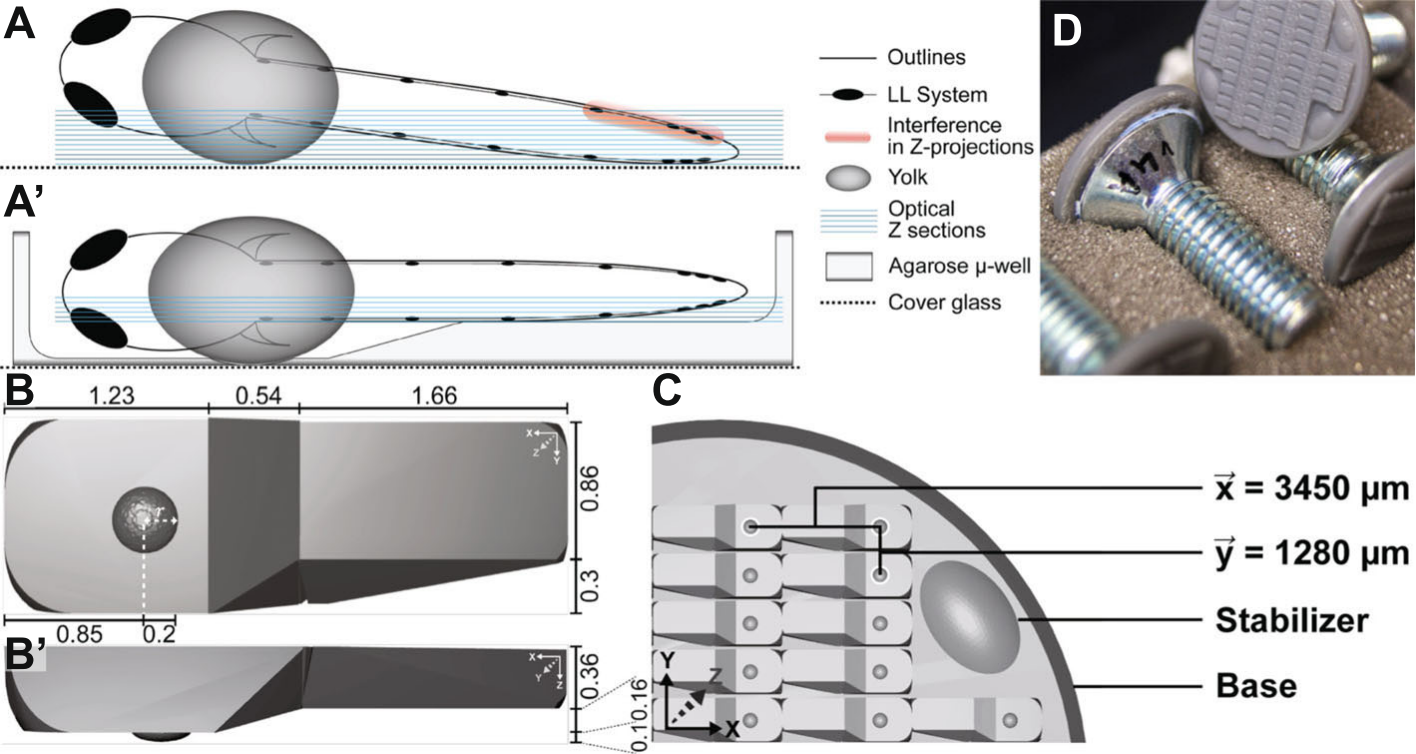
Stamp and μ-well dimensions (**A, A’**) Scheme showing a zebrafish embryo mounted classically, directly on the coverslip (**A**) and with μ-well (**A’**). **B, B**′ Top-view (**B**) and lateral view (**B**′) of a single μ-well with dimensions in mm. **C** Design and dimensions of the stamp. The base connects all parts and fits exactly a Ø 20 mm coverslip of a Ø 35 mm imaging dish. $\overrightarrow{x}$ and $\overrightarrow{y}$ are the distances between two adjacent μ-wells. Stabilizers were introduced as cornerstones and to make the structure more rigid. **D** For better handling, the stamp is mounted onto a countersunk screw

Therefore, there is a need for methods to standardize sample mounting and image acquisition of multiple embryos at a spatiotemporal resolution suited for 3D segmentation and 2D tracking experiments. The protocol we describe here was designed to be used with XY scanning universal sample holders that usually come with any motorized-stage inverted microscope. Similar to previous approaches [14–17], it uses a 3D-printed stamping device to produce an agarose imprint with a diameter of 20 mm on the cover glass of a 35 mm μ-dish. The imprint consists of 44 equally spaced micro-wells (μ-wells), which are designed to fit the average morphology of a zebrafish embryo between 24 and 96 h-post-fertilization (hpf) (Fig. 1a-b).

The aim was to develop a standardized mounting method allowing us to: (i) mount many samples in parallel in a 2D coordinate system of rows and columns, (ii) reduce the acquisition time and thus photo-bleaching and photo-toxicity during imaging, (iii) semi-automatize the acquisition, (iv) reduce the post-processing steps, and (v) facilitate subsequent processing such as genotyping due to a 1:1 correlation between image data and specimen arrangement sequence.

## Results

**Fig. 2:**
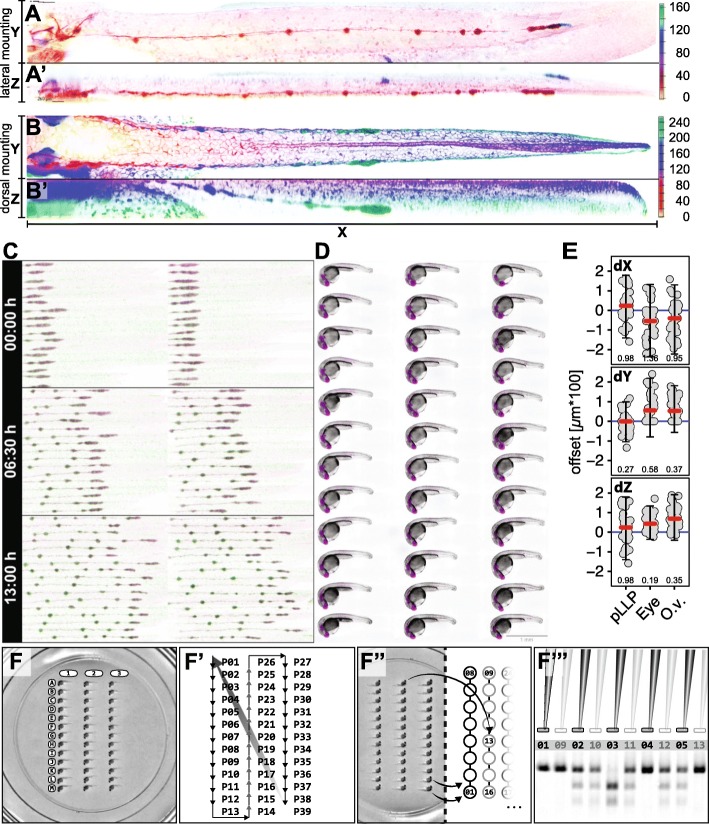
Representative Results. **A** Maximum intensity projection (MIP) of a 50 hpf embryo mounted on its side (XY) and corresponding MIP of the orthogonal view (XZ) of the same embryo showing how flat the embryos are mounted in the plate (**A’**). **B** MIP of a 40 hpf embryo mounted dorsally (XY) and corresponding MIP of the orthogonal view (XZ) of the same embryo (**B′**). Depth colour encoding is indicated by colour scales on the right. **c** Multi-position (36), multi-channel (2) time-lapse recording (13 h duration; 15 min. Interval, see also Additional file 1: Movie S1). **D** Multichannel (2) Extended Depth of Focus (EDF) projections from widefield Z-stacks (recorded with 20x Objective). Scale Bar = 1 mm (**E**) Multi-point coordinates in X, Y and Z (recorded with 40x Objective). The *offset* describes the distance of each point from the mean of all points in X, Y and Z (See methods). Panel 1–3 (top to bottom) show dimensions X, Y and Z in comparison for the pLLP, the eye and the otic vesicle. The red line indicates the median, the blue line indicates zero offset, error bars indicate mean ± standard deviation. Numeric values indicate the variance. **F-F**″’ Systematic retrieval for genotyping. **F** Mounted embryos in a 2-D coordinate system of rows (A-M) and columns (1–3). **F**′ Imaging Sequence in a snake by column fashion. In a time-lapse setting, it starts at point 1 (P01) again to initiate the next timepoint. **F**″ After imaging, the embryos are retrieved in the same sequence as they were imaged (snake by column, left panel). **F**”’ Each genotyping result on the electrophoresis gel is easily correlated to one imaging dataset with defined X-Y coordinates

Our 3D-printed stamp improves two main aspects of specimen preparation: orientation in Z (Figs. 1a-a’, 2a-a’) and standardization of the embryo arrangements in X and Y (Figs. 1c, and 2d). To demonstrate the efficacy of this method, we used the zebrafish posterior lateral line (pLL) system as a model.

The lateral line is a mechano-sensory organ that enables aquatic vertebrates to sense water movements and pressure, and therefore receive information about their environment. Its functional units are the neuromasts (NMs), which are groups of about 30 cells organized around a core of hair cells surrounded by support cells [18]. In the adult, the lateral line comprises hundreds of NMs distributed over the entire body surface. The first NMs to form in the zebrafish embryo are deposited by two groups of about 130 epithelial cells, the posterior lateral line primordia (pLLP), which migrate from head to tail on both sides of the embryo during the second day of development (Fig. 2c, Additional file 1: Movie S1). At about 50 hpf the pLL spreads linearly along the lateral body flanks of the embryo (Fig. 2a-b’; ~ 2 mm in length). Using this mounting technique, lateral line development can be recorded over more than 20 h, in up to 44 positions, in a confocal Z-stack of less than 120 μm and a time interval of 5–10 min (depending on the number of channels and exposure time).

Each morphogenetic process demands unique imaging conditions and may present several challenges that have to be overcome. Table 2 highlights specific challenges concerning imaging of the lateral line development.

**Table 2:** Specific challenges when imaging pLLP morphogenesis.

Challenges	Solutions
pLL morphogenesis lasts about 30 h: Embryos need to be immobilized but still survive and grow over a longtime period	Use of low concentration of LMPA containing Tricaine
pLLP migrates over almost 2 mm so the area to image is 2 to 3 times longer than one field of view (FOV): (i) If the embryos are not mounted parallel to the coverslip, the size of Z-stack increases significantly. (ii) The contra-lateral pLLP eventually enters the Z-volume and introduce interfering signal in post-processing Z-projections.	Multiple FOV need to be imaged and stitched The use of μ-wells with a defined shape orients the tails parallel to the coverslip (see Fig. 2a)' to both reduce the size of Z-stacks and prevent the interference of the contra-lateral pLLP.

Figure 2c shows three timepoints of a single time-lapse recording with 34 positions, 2 stitched FOVs, 2 channels (transgenes *cldnb:GFP* and *cxcr4b:H2B-RFP*), imaged with a 10 min. interval over a period of 13 h (Additional file 1: Movie S1). As mentioned above, the most crucial factor for automation of zebrafish embryo imaging is (i) that the embryos are arranged at equidistant spaces and (ii) that they are oriented in the same way. Using our mounting protocol allows the user to image zebrafish embryos systematically with up to 5 times more embryos as compared to traditional mounting methods. Figure 2d shows 36 embryos in 2 channels (brightfield + transgene *cldnb:GFP*) imaged with a 10x objective after an overnight time-lapse, highlighting (1) the regularity in mounting and (2) embryo integrity after time-lapse imaging (see also Additional file 1: Movie S1). To quantify mounting precision and reproducibility, the positions (in X, Y and Z) of three common zebrafish model organs (i) the pLLP, (ii) the eye and (iii) the otic vesicle were acquired and evaluated (Fig. 2e, see methods ‘offset calculation’).

## Discussion

It has been more than 10 years since zebrafish has been recognized as a very powerful model organism for in vivo imaging and high content screening [9, 19]. Although efforts have been made to standardize zebrafish embryo mounting and imaging, most protocols were designed for high throughput widefield screening [16, 17, 20, 21] rather than high-resolution confocal microscopy [22–24]. In recent years a couple of studies tackled specifically this issue by developing standardized mounting methods using 3D-printed stamps [14, 15, 25, 26]. Here we provide a handy and simple approach for a standardized and much more efficient way of mounting zebrafish for high resolution confocal microscopy.

### Troubleshooting

The existing limitations in classical mounting methods, the solutions proposed by our new method and the improvements are summarized in Table 1. The most critical steps of the protocol are:

i. The detachment of the stamp from the solidified 1% Agarose (see ‘Design and preparation of 3D stamp’), which can cause the formation of air inclusions between the cover glass and the agarose.
ii. The speed of polymerization of the 0,3% LMPA which should not be too fast to give enough time to place and orient each embryo in a μ-well.
iii. The application of the 0,5% LMPA to the embryos embedded in 0,3% LMPA (see ‘Mounting’ part of the Methods section) which should happen very carefully so that the embryos are not carried away.

### Major improvements

Our method presents two major improvements over currently used mounting methods:

1. The low percentage LMPA used allows for:
  - An extended timespan for mounting which is necessary to align the high number of embryos.
  - Facilitated retrieval of the embryos after imaging and systematic identification of individual positions (e.g. for genotyping). For the latter, make sure your numbering for identification is in accordance with the sequence in which you imaged the embryos (Fig. 2f-f”’).
  - More freedom for the embryo to grow and elongate during long time-lapse imaging (Fig. 2c).

2) The mounting cast allows for:
  - Standardized and reproducible positions of the embryos as shown for the lateral line primordium, the eye and the otic vesicle (Fig. 2e).
  - Significant increase of number of embryos (more than 5 times more) imaged in a single experiment (Fig. 2c-d).
  - Consistent and minimized distances between different positions (Fig. 1c)
  - Identical and stage-aligned orientations of all embryos in XYZ (Figs. 1, and 2d-e and 5)
  - Significant reduction of the Z-stack size and therefore of the illumination of the samples (Fig. 2a’, e).

### Flexibility

The design of the μ-well is not limited to a specific model organism or spatial orientation but different μ-wells may be designed exactly fitting the specific requirements. In fact, we have developed and successfully tested a stamp for dorsal mounting (Fig. 2b, b’ – dorsal mounting) of zebrafish larvae. Also, it is possible to adopt the existing models for mounting other embryos including *O.latipes* (Medaka). All stamp models developed can be found at Github (https://github.com/LecaudeyLab/3DModels) and are posted as a technology offer at INNOVECTIS (http://innovectis.de/en/technologies/goethe-depository/3d-printed-stamp-for-standardized-mounting-and-high-content-confocal-imaging-of-zebrafish-embryos/) to ease transfer of technology.

### Further improvements

While round dishes require a manual adjustment to align the embryo body axes to the microscope stage axes, using rectangular dishes together with a rectangular stamp would minimize the degrees of freedom when mounting the dish onto the sample holder.

### Comparison to existing methods

In the past 5–10 years, several labs have developed methods for high-throughput imaging based on microfluidic devices. The limitations of such approaches is (i) that it is difficult and expensive to develop for non-specialized labs and (ii) it is perfectly suited for high-throughput screening, low-resolution purposes but less for long time-lapse or high resolution imaging since the embryos are generally not immobilized [20, 21, 27, 28]. Approaches based on 3D-printed stamps, like ours, are much more flexible and easier to establish in any lab, either using an in-house 3D printer or as we did it by outsourcing the printing at low costs. Recently, a multi-sample preparation protocol for long-term time-lapse imaging using a light-sheet fluorescence microscope (LSFM) has been published [29]. However the amount of embryos that can be imaged simultaneously was limited to five due to the specific sample mounting imposed by the orthogonal arrangement of the objectives in such systems. In principle, our 3D-stamp could however be combined with more recently-developed LSFM that are not based on orthogonal objectives and thus can use classical cover slip or petri dishes mounting method [30].

## Conclusions

In conclusion, the main advantages of our 3D-printed stamp over existing ones are:

(i) the amount and the narrow arrangement of μ-wells allow for reduced stage movements and imaging of many embryos simultaneously (up to 44 in our case).
(ii) improved time resolution during time lapse imaging of multiple positions.
(iii) the specifically-designed form of the μ-well maintains the AP axis of the embryo parallel to the coverslip and thus allows to reduce Z-stack size, exposure time, photo-bleaching and photo-toxicity, and image reorientation post-processing and to improve signal-to-noise ratio.
(iv) the reduced concentration of LMPA to 0.3% gives the embryo more freedom to grow and change its shape [14, 15] while still being sufficiently immobilized.
(v) semi-automated imaging and further downstream sample processing, including genotyping significantly facilitated by the organization in a 2D coordinate system.

## Methods

### Zebrafish husbandry and transgenic lines

Animals used in this study were obtained from own breeding at the animal facility of the Goethe University Frankfurt according to the German animal welfare act and approved by the German authorities (veterinary department of the Regional Board of Darmstadt). Adult zebrafish were maintained under standard conditions and embryos were staged according to Kimmel et al. (1995). The Transgenic lines *Tg(− 8.0cldnb:lynEGFP)*^*zf106*^ (*cldnb:gfp*) and *TgBAC (cxcr4b:H2B-RFP)*^*fu13*^ (*cxcr4b:H2B-RFP*) have been described previously [31, 32].

### Design and preparation of 3D-stamp

To model a negative of an average zebrafish embryo between 24 and 96 hpf, the dimensions of different embryonic structures were measured on corresponding stage-match embryos:

(i) Embryo: Length / width (X, Y)
(ii) Yolk Sac: Diameter (in XY) and depth (in Z)
(iii) Trunk: Length / width / depth (X, Y, Z)

Using Microsofts ‘3D Builder’, a single μ-well was assembled by transformation of basic shapes like cube, sphere and wedge. Afterwards, the maximum number of μ-wells fitting in a circle with a diameter of 20 mm were positioned. The 3D printing was then carried out by a commercial partner using a Formlabs ‘Form1+’ extrusion printer with the Formlabs material ‘Tough’ photopolymer. Using this material, it is possible to print structures at a maximum resolution of 25x50x100 μm, which is necessary to capture the intricate details of the embryonic morphology. To ease the handling, a zinc-plated countersunk screw with dimensions 8 × 20 mm (DIN7991) was attached to the back of the printout using super-glue (Fig. 1d).

### Preparation of the agarose mounting cast

**Fig. 3:**
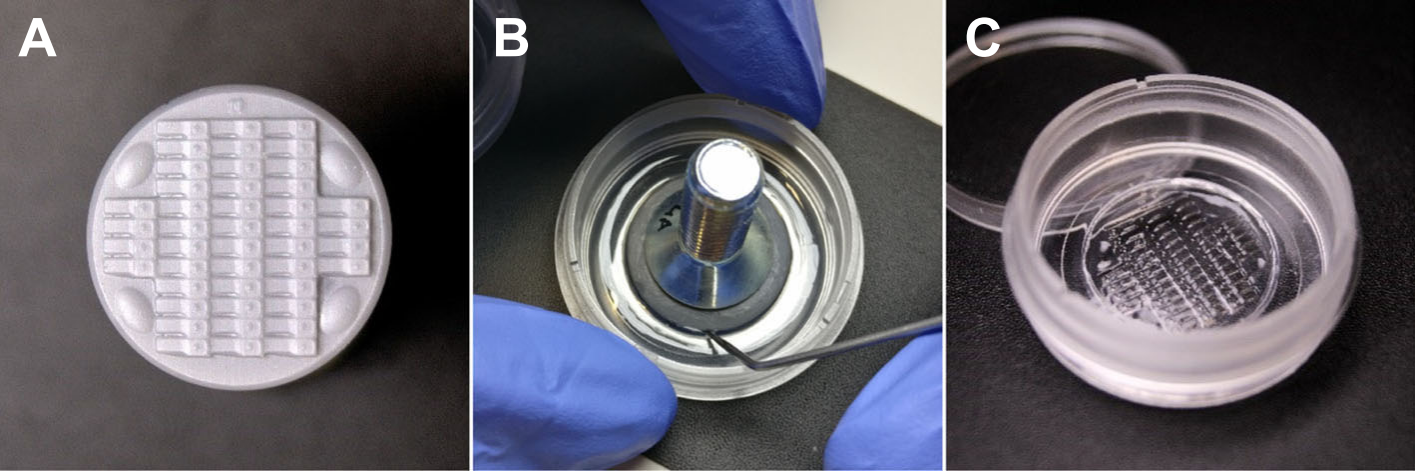
Stamping procedure. **a** Clean stamp surface **b** Removal of the 3D-stamp from the polymerized agarose cast using a preparation needle **c** Ready-to-use mounting cast

Stamps were cleaned of dust or other particles with 70% Ethanol and pressured air. A solution of 1% agarose (w/v) was prepared in a clean 100 mL blue cap bottle by dissolving 200 mg of agarose in 20 mL of E3 in the microwave. 650 μL of this solution was then applied with a 1 mL pipette to the coverslip (Ø 20–21 mm) of a 35-mm imaging dish (e.g. ibidi μ-dish or MatTek microwell, see list of materials). The stamp was gently placed on it (Fig. 3a-b) and its position carefully adjusted to be in the centre of the coverslip. The dish was then gently rotated to distribute the excess agarose over the entire dish surface to stabilize the imprint once polymerized.

► If bubbles emerge upon immersion of the pipette tip into the 1% agarose solution, the solution is too hot. Wait until no or almost no bubbles are appearing anymore.

The agarose was then allowed to polymerize for ~ 30 min. To remove the stamp, a clean preparation needle was slipped between the stamp and the polymerized agarose (Fig. 3b), and the stamp was then removed by holding the dish in place and carefully but swiftly lifting it. If necessary, air bubbles appearing between the cover glass and the agarose imprint were eliminated by puncturing with a preparation needle. The mounting cast (Fig. 3c) was either used immediately or stored at 4 °C for several days (with closed lid).

### Preparation of mounting media

Two solutions of low-melting point agarose (LMPA) were prepared in clean 100 mL blue cap bottles by dissolving 60 mg and 100 mg LMPA in 16 mL of E3 in the microwave (0,375% and 0,625%, respectively). Per stamped cast, 2 aliquots of 1.6 mL were prepared in 2 mL tubes for each LMPA concentration, and placed in a heating block adjusted to 41 °C. For live imaging, 400 μL of 4,2 mg/mL Tricaine (25X) were added to keep the embryos anesthetized during the imaging session. Final concentrations of LMPA were therefore 0,3% and 0,5%, respectively. The LMPA containing Tricaine was prepared fresh for each mounting session.

### Mounting

**Fig. 4:**
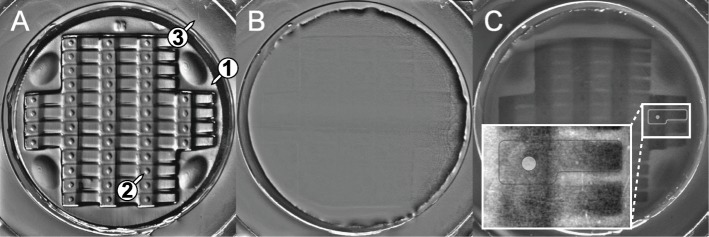
Mounting cast under the stereo microscope (**a**) without LMPA and (**b, c**) with LMPA with different mirror and contrast adjustment in B (μ-wells not visible) and C (μ-wells visible) (1). inner well and mounting area (2) single μ-well (3) outer well. See also movie S2

Before mounting, the quality of the imprint was examined under a stereo-microscope and deformation of single μ-well were corrected with a preparation needle, if necessary. Since the outlines of the stamp become transparent when adding the LMPA and to be able to still locate the embryos for alignment, the illumination contrast and mirror of the transmitted light base was adjusted to see the μ-wells again (Fig. 4a-c and Additional file 2: Movie S2).

The embryos were anesthetized in the petri dish they grew in with 4 or 5 drops of 4,2 mg/mL Tricaine (40 μg/mL in E3) added 4–5 min before usage. For mounting, the imprint was first gently filled from the border (Fig. 4a and Additional file 2: Movie S2) with 500 μL of 0.3% LMPA. Then, 44 embryos (for a 44 μ-well stamp) were collected from their petri dish with a glass Pasteur pipette (Additional file 2: Movie S2). To minimize the amount of liquid added to the LMPA, the embryos were allowed to sink to the air – liquid interface and immediately added in one drop to the liquid LMPA in the stamped cast.

**Fig. 5:**
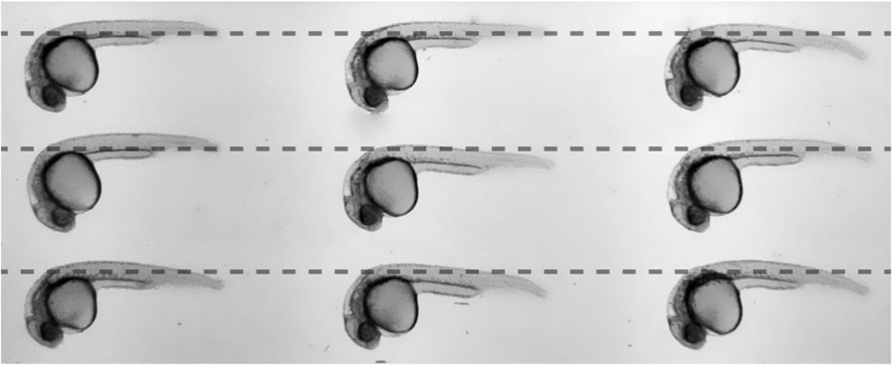
Mounted and tail-aligned embryos (9 out of 44). Stripe lines indicate horizontal tail alignment

Each embryo was moved to a separate μ-well with a preparation needle. The yolk was positioned within the half-spherical structure of each well, and the tail aligned horizontally with the shape of the well (Fig. 5 and Movie S2). The LMPA was allowed to polymerize for about 40 min. For time-lapse recording longer than 1 h, 1 mL of 0.5% LMPA was carefully added on top and allowed to polymerize for another 10 min to stabilize the structure.

The speed of polymerization of the LMPA is critical and should not be too fast to give enough time to place and orient each embryo in a μ-well. The temperature of the room should be around 23 °C. For indefinite time of embryo orientation, higher room temperature or a 5 V terrarium heating mat (at maximum temperature, ca. 38 °C) can be used. For this, a hole with the diameter of an imaging dish should first be cut in the middle of the heating mat. For mounting, the mat should be placed on the stereomicroscope stage with the dish in the hole.

Extra attention should be given when adding 0.5% LMPA on top of the 0.3% LMPA for time-lapse imaging. The LMPA will still be very fragile. Apply the 0.5% LMPA to the outer well first, then carefully raise the level.

### Imaging setup

**Fig. 6:**
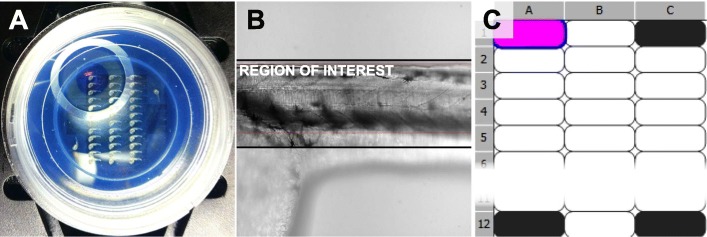
Imaging setup. **a** Stage positioned on embryo at position 1 (P01 – upper left corner) **b** ROI definition and focus refinement in brightfield. **c** Navigation through the μ-wells by a custom defined well plate. Magenta well is current. Black wells are used to calibrate the well plate

The dish was placed onto the sample holder of an inverted confocal spinning disc microscope so that the embryos aligned to the Y axis of the microscope stage. The stage was then moved to place the embryo at Position 01 (P01, top-left position) right above the objective (Fig. 6a).

The following steps describe the procedure on a fully motorized Nikon-Ti spinning disc system using NIS5.0, but can be performed with any commercial software:

1. Define positions (option **a** or **b**)
  - Pre-defined points list in XY
    Move the stage to P01 (Fig. 6a)
    Define multipoint list (‘Custom Multipoint Definition’ in NIS 5.0) using the fixed distances (dX and dY) between embryos
    dX/dY = 3450 / 1280 μm
    Bring P01 in focus and offset all points in Z
    Save the list to be able to re-use it in a future experiment.

To re-use a pre-existing point list: load the list, move to P01, bring it in focus and offset all points in X, Y and Z to align all points to reference P01.

b. Custom well plate
  Define a new custom well plate (Fig. 6C)
  Calibrate the stage
  Move to each position

2. Refine Positions
  Move to the single positions one after another. If necessary, refine X, Y and Z and update the coordinates.
3. Start imaging

### Retrieval

**Fig. 7:**
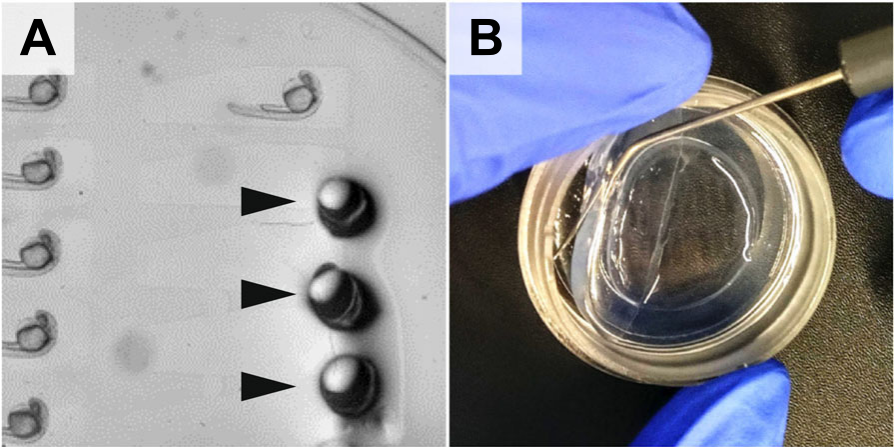
Embryo retrieval. **a** Arrowheads point to incisions indicating locations where the pipette was introduced into the agarose to retrieve the embryos **b** Removal of the agarose bed with a preparation needle

For further experiments such as genotyping, the embryos were retrieved from the agarose in the same sequence as they were imaged (snake by column, Fig. 2f-f”). For this purpose, a glass pipette was directed to the head region of an embryo and a gentle under pressure was applied till the embryo was gently sucked into the glass pipette (Fig. 7a). Each embryo was then placed in a single tube of an 8-tube PCR strip and lysed to extract genomic DNA. The genotyping PCR was then performed and analysed by gel electrophoresis using an 8x-multichannel pipette. When using a 34-well comb, the pipette tips will reach every second well of the agarose gel. Filling the wells staggered (offset by 1), one can load 4 × 8 wells in one row (Fig. 2f”’). Since each embryo has a defined position, it is straightforward to associate each genotype to the corresponding image data (Fig. 2f-f”’). Since a single mismatch would mess up the entire experiment by resulting in a frameshift of the one-to-one correspondence, this is a very important feature. The imaging dish can be reused several times. For cleaning remove the agarose bed from the dish using a small scoop or preparation needle (Fig. 7b) and wipe it gently with a lint-free tissue soaked in ethanol.

### Offset calculations

Multi-point coordinates of three independent experiments, each with information of 39 points and coordinates in X, Y and Z, were saved and used to demonstrate mounting precision (Additional file 3: Dataset S3). For each set of 39 points the global means of X, Y and Z were calculated and used as references for distance calculation. For graphical representation ggplot2 for R was used [33].

1. Calculate global means

$$ Reference(X)={\upmu}_{\mathrm{X}}={\mathbf{X}}_{\mathbf{0}} $$

$$ Reference(Y)={\upmu}_{\mathrm{Y}}={\mathbf{Y}}_{\mathbf{0}} $$

$$ Reference(Z)={\upmu}_{\mathrm{Z}}={\mathbf{Z}}_{\mathbf{0}} $$

2. Calculate Offset

$$ \boldsymbol{Offset}{\left(\mathrm{X}\right)}_{\mathrm{n}}={\mathrm{X}}_{\mathrm{n}}-{\mathrm{X}}_0 $$

$$ \boldsymbol{Offset}{\left(\mathrm{Y}\right)}_{\mathrm{n}}={\mathrm{Y}}_{\mathrm{n}}-{\mathrm{Y}}_0 $$

$$ \boldsymbol{Offset}{\left(\mathrm{Z}\right)}_{\mathrm{n}}={\mathrm{Z}}_{\mathrm{n}}-{\mathrm{Z}}_0 $$

## Materials

**Table:** 

Description	Company	Catalog No.
μ-Dish	Ibidi	81,218–200
Stamp	Printed with a Formlabs ‘Form1+’ 3D printer. Resin = Formlabs ‘Tough’ photopolymer. Resolution = 25x50x100 μm	–
Preparation needles	VWR	USBE5470
Pasteur Pipettes	Roth	4518
Rubber or silicone bulb	VWR	612–2327
Microtubes 2 mL	Sarstedt	2691
Heating block	PeqLab	HX2
Microwave oven	Severin	MW7849
Stereo microscope	Leica	M165FC
Transmitted Light Base	Leica	MDG36
Agarose	Sigma	A9539
Low-melt Agarose	Roth	6351.2
16% Formaldehyde (w/v), Methanol-free	Thermo Fischer	28,908
Tricaine methane sulfonate	Sigma	A5040
NaCl	Roth	9265.2
KCl	Roth	P017.1
CaCl_2_	Roth	886.1
MgSO_4_*7H_2_0	Roth	T888.2
Countersunk screw DIN7991, 8 × 20 mm	Dresselhaus (Hornbach)	7,662,389
Superglue Blitzschnell Pipette	UHU (bueroshop24)	509,141
10X P-APO ƛ/0.45/4.0	Nikon	MRD00105
20X APO LWD ƛ-S/0.95/0.95	Nikon	MRD77200
40X APO LWD ƛ-S/1.15/0.6	Nikon	MRD77410
Eclipse Ti-E	Nikon	MEA53100
Motorized XY-table	Nikon	MHTIMOT-E
Piezo Z-Table (300 μm range)	Nikon	MHPIEZOZT
Universal Sample Holder	Nikon	MH00555001020
CSU-W1	Nikon	BIO1
ZYLA Plus	Nikon	BIO4

## Supplementary information

: **Additional file 1: Movie S1.** Multi-position time-lapse. The movie shows a multi-position, multi-timepoint (15 h / 10 min. interval) dual-channel Z-projection of confocal-Z-stacks of about 50 slices (2.5 μm spacing). Two fields of view were stitched per embryo.

: **Additional file 2: Movie S2.** Mounting procedure. The video is divided in 5 consecutive episodes. (E1) Add mounting medium to the agarose cast (E2) Adjust Light path to make the μ-wells visible again (E3) Move mounting dish aside and collect embryos (E4) Apply embryos to the centre of the mounting dish (5) Distribute embryos.

: **Additional file 3: Dataset S3.** Multi-point coordinates. Single coordinates represent the centre of each respective organ in X, Y and Z as perceived by using *cldnb:lyn-gfp* transgenic embryos. The data is provided as ‘.xml’ files, the native points-list data format of NIS-Elements. To parse the files and process the data R was used [34] [35]. The documentation with appropriate code comments is presented as ‘.html and ‘. Rmd’ files.

## Data Availability

All data generated or analysed during this study are included in this published article and its supplementary information files.

## Abbreviations

dX: Distance in X
dY: Distance in Y
E1–5: Episode 1–5
EDF: Extended Depth of Focus
FOV: Field of View
hpf: Hours post fertilization
LMPA: Low-melting point agarose
LSFM: Light-sheet fluorescence microscope
MIP: Maximum Intensity Projection
NM: Neuromast
P01: Position 1
pLL: Posterior lateral line
pLLP: Posterior lateral line primordium
SNR: Signal-to-Noise Ratio
μ-well: Micro-well
