# Supplementary figures and images for: Standardized mounting method of (zebrafish) embryos using a 3D-printed stamp for high-content, semi-automated confocal imaging
Source: BMC Biotechnol. 2019 Oct 22;19:68. doi: 10.1186/s12896-019-0558-y (PMC6805687; doi:10.1186/s12896-019-0558-y)

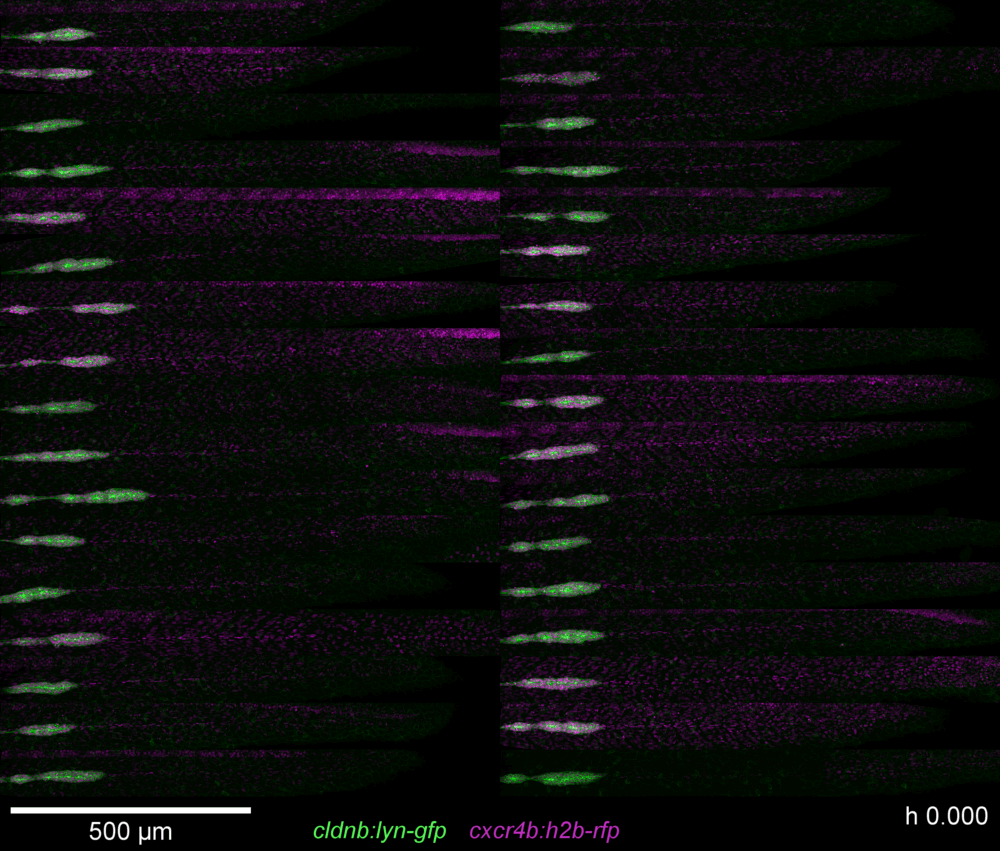

Supplement: Supplementary file 1 — : Additional file 1: Movie S1. Multi-position time-lapse. The movie shows a multi-position, multi-timepoint (15 h / 10 min. interval) dual-channel Z-projection of confocal-Z-stacks of about 50 slices (2.5 μm spacing). Two fields of view were stitched per embryo. [file 12896_2019_558_MOESM1_ESM.gif]
